# Gravity Water Waves over Constant Vorticity Flows: From Laminar Flows to Touching Waves

**DOI:** 10.1007/s42286-025-00127-4

**Published:** 2025-10-27

**Authors:** Francisco Gonçalves

**Affiliations:** Delft Institute of Applied Mathematics, Delft University of Technology, P.O. Box 5031, Delft, 2600 GA The Netherlands

**Keywords:** Free surface waves, Periodic traveling, Constant vorticity, Overhanging, Touching, Crapper, Critical layers

## Abstract

In a recent paper, Hur and Wheeler (J Fluid Mech 896:1, 2020) proved the existence of periodic steady water waves over an infinitely deep, two-dimensional and constant vorticity flow under the influence of gravity. These solutions include overhanging wave profiles, some of which exhibit surfaces that touch at a point and thereby enclose a bubble of air. We extend these results by formulating a problem that encompasses both infinitely deep and finitely deep flows, and by proving the existence of a continuous curve of water waves that connects a laminar flow to a touching wave for fixed, nonzero gravity. This implies the existence of a wave profile featuring a vertical tangent at a point, which is not overhanging, and is referred to as a breaking wave. We also study the behavior of critical layers, which are points where the horizontal velocity vanishes, near the surface. In particular, this result holds for arbitrary vorticity.

## Introduction

We study periodic water waves traveling over a two-dimensional, incompressible, inviscid flow with constant vorticity. The effect of gravity is included, while surface tension is neglected. We prove the existence of waves with an overhanging profile for flows of finite depth, including profiles whose surfaces touch at a point, referred to as touching waves. Furthermore, for both finite and infinite depth, and for fixed nonzero gravity, we establish the existence of a continuous curve of solutions connecting a laminar flow to a touching wave.

In 1957, Crapper [2] discovered an explicit and smooth curve of exact solutions for capillary waves propagating in an irrotational flow in the absence of gravity. As the amplitude increases, the crests of Crapper’s solutions become flatter while the troughs become sharper. This behavior contrasts with that of gravity waves. The endpoint of Crapper’s curve is a touching wave. Akers, Ambrose, and Wright [3], as well as de Boeck [4], constructed small-amplitude gravity waves close to Crapper’s solutions. Córdoba, Enciso, and Grubic [5] further extended this work by constructing a touching wave.

In the setting of constant vorticity with zero surface tension and zero gravity, numerical studies by Dyachenko and Hur [6, 7] and Hur and Vanden-Broeck [8] found evidence of explicit solutions with the same wave profile as Crapper’s solutions. Hur and Wheeler [9] later established the existence of such solutions analytically. More recently, Crowdy [10] showed that these solutions can be understood as part of a broader framework of explicit solutions in the constant vorticity case under the same assumptions.

Using an Implicit Function Theorem argument, Hur & Wheeler [1] constructed overhanging and touching waves for small gravitational effects in a constant vorticity flow. In this paper, we take these matters further, by considering both finitely and infinitely deep flows, and by applying local bifurcation theory and compactness arguments to improve upon their results. This leads to a smooth curve of solutions from the laminar flow up to a touching wave for fixed small nonzero gravity. Along this curve, we obtain waves whose profiles are vertical at a point but never overhanging, referred to as breaking waves.

We also analyze the local behavior of critical layers (points where the horizontal velocity of the fluid vanishes) at the surface of breaking and overhanging waves and how they interact with gravity. In particular, this result holds for arbitrary vorticity.

We begin by introducing and reformulating the water wave problem in Sect. 2. This is followed by an extension of the result of Hur and Wheeler to the finite depth case, presented in Sect. 3. Next, in Sect. 4, we construct a continuous curve of solutions starting from the laminar flow and continuing to the touching wave. Finally, Sect. 5 is devoted to analyzing the local behavior of critical layers at points where the wave has a vertical tangent.

## Preliminaries and Reformulation

We consider the water wave problem for a periodic, steady wave traveling over a two-dimensional, incompressible, inviscid fluid under the influence of gravity while neglecting surface tension. For simplicity, we assume the fluid has unit density. In Cartesian coordinates, we assume the *x*-axis points in the direction of wave propagation, while gravity acts in the negative *y*-direction. We consider a moving frame of reference so that the flow is stationary. Suppose the flow occupies a region *D* in the *x*, *y* plane and is bounded from above by a free surface *S*. Using a stream function, $\psi$, we can express this problem as

1a $$\begin{aligned}&\!\bigtriangleup \psi =-\omega&\text {in }D, \end{aligned}$$

1b $$\begin{aligned}&\psi =0&\text {on }S, \end{aligned}$$

1c $$\begin{aligned}&\frac{1}{2}|\nabla \psi |^2+ g y= b&\text {on }S, \end{aligned}$$

where $\omega$ denotes the vorticity, which is assumed to be constant in *D*. Moreover, *g* denotes the gravitational constant and *b* is used to denote a Bernoulli constant. Note that *S* is a free boundary, (1b) and (1c) are, respectively, the kinematic and dynamic boundary conditions. We assume that both *D* and $\psi$ are $\tfrac{2\pi }{k}$ periodic in the *x* direction and symmetric with respect to the vertical lines beneath the wave crest and trough. In addition, we consider two alternative boundary conditions at the bottom:

2a $$\begin{aligned}&\nabla \psi -(0,-\omega y-c)\rightarrow (0,0)&\text {as }y\rightarrow -\infty , \end{aligned}$$

2b $$\begin{aligned}&\psi =q&\text {on }y=-h, \end{aligned}$$

where *c* denotes the wave speed and *q* is an a priori unknown constant. Note that in the finite depth case, the wave is also assumed to travel with speed *c*. Equation (2a) corresponds to the infinite depth case, where *D* is unbounded from below and (2b) corresponds to the finite depth case, where *D* is bounded from below by $\{y=-h\}$, where *h* is a positive constant. In fact, in the finite depth case, the problem is invariant under a vertical translation after a suitable modification of *B*.

We now make the change of variables

$$\begin{aligned} x\rightarrow kx, \qquad \qquad y\rightarrow ky,\qquad \qquad \psi \rightarrow \frac{k}{c}\psi \end{aligned}$$

and introduce the dimensionless parameters

$$\begin{aligned} \Omega =\frac{\omega }{ck},\qquad G=\frac{g}{kc^2},\qquad B=\frac{b}{c^2 }, \qquad H=-hk,\qquad Q=\frac{qc}{k}. \end{aligned}$$

In these non-dimensional variables, (1) becomes

3a $$\begin{aligned}&\!\bigtriangleup \psi =-\Omega&\text {in }D, \end{aligned}$$

3b $$\begin{aligned}&\psi =0&\text {on }S, \end{aligned}$$

3c $$\begin{aligned}&\frac{1}{2}|\nabla \psi |^2+ G y= B&\text {on }S \end{aligned}$$

and the bottom boundary conditions (2) become

4a $$\begin{aligned}&\nabla \psi -(0,-\Omega y-1)\rightarrow (0,0)&\text {as }y\rightarrow -\infty , \end{aligned}$$

4b $$\begin{aligned}&\psi =Q&\text {on }y=-H. \end{aligned}$$

This completes the non-dimensional formulation of the problem. In the next section, we reformulate it using tools from complex analysis.

### Reformulation

For any $d>0$ possibly infinity, we define

$$\begin{aligned} \mathbb {S}_d:= \{\alpha +i\beta \in \mathbb {C};0>\beta >-d\}. \end{aligned}$$

#### Lemma 1

Consider a $2\pi$ periodic domain *D* that is bounded from above by *S* and is either unbounded from below or bounded by $\{y=-H\}$. Then there exists a unique *d* and a conformal map *z* from $\mathbb {S}_d$ onto *D* satisfying the following conditions.

(i) *z* extends continuously to $\{\beta =0\}$ and maps $\{\beta =0\}$ to *S*;
(ii) $x(\alpha +i\beta )-\alpha$ and $y(\alpha +i\beta )$ are $2\pi$ periodic functions of $\alpha$;
(iii) if $H<\infty$ then $d<\infty$ and the continuous extension of *z* maps $\mathbb {S}_d\cup \{\beta =-d\}$ to $D\cup \{y=-H\}$ continuously;
(iv) if $H=\infty$ then $d=\infty$ and $z(\alpha +i\beta )-(\alpha +i\beta )\rightarrow 0 \text { as } \beta \rightarrow -\infty .$

where $x(\alpha +i\beta )$ and $y(\alpha +i\beta )$ denote the real and imaginary parts of $z(\alpha +i\beta )$, respectively.

For a proof of the finite-depth case, see the appendix of [11]. The proof for the infinite-depth case is very similar.

We now state a result concerning the analyticity of the free surface *S* and the analytic extension of the stream function $\psi$. Constantin and Escher [12] established a primary result on the analyticity of the free surface under the assumptions that the fluid contains no stagnation points and that the surface can be represented as the graph of a function. Notably, their analysis permits the vorticity to be a real analytic function. Later, Aasen and Varholm [13, Theorem 2.5] improved this result for the affine vorticity case, showing that the no-stagnation assumption is required only on the boundary. Our proof follows the strategy of [13] and uses a local argument that accommodates free surfaces that are not representable as single-valued graphs.

#### Proposition 2

Let *z* be the conformal map obtained in Lemma 1 and let $\psi$ be a solution of (3). Assume that the free surface *S* is a $C^1$ curve and that $z_\alpha$ has no zeros on the set $\{\beta =0\}$. Then the following three properties hold.

(i) The free surface *S* is real analytic;
(ii) The map *z* has a complex analytic extension to an open neighborhood of $\mathbb {S}_d\cup \{\beta =0\}$;
(iii) The stream function $\psi$ has a real analytic extension that satisfies $\bigtriangleup \psi =-\Omega$ to an open neighborhood of $\mathbb {S}_d\cup \{\beta =0\}$.

#### Proof

(i) Let *p* be an arbitrary point on *S*. There exists an open set *U* contained in *D*, such that $p\in \partial U \cap S$ and $S\cap U$ can be represented either as $\left( x,\eta (x)\right)$ or $\left( \eta (y),y\right)$ for some function $\eta$. Applying [14, Theorem 2] with *u* as the stream function and *g* as the dynamic boundary condition. Note here the assumption of $z_\alpha \ne 0$ on the boundary is equivalent to $\nabla \psi \ne 0$ on the boundary. This yields that $\partial U \cap S$ is analytic. Since *p* was arbitrary, it follows that *S* is real analytic.
(ii) This follows from applying [15, Theorem 2.2, page 299].
(iii) Since $\bigtriangleup \psi +\Omega =0$ in *D* and $\psi =0$ on the real analytic curve *S*, we can apply [16, Theorem A] to yield the claim.

Proposition 2 ensures that both the free surface and the associated stream function extend analytically to nearby regions, this is necessary for studying the local behavior of critical layers in Sect. 5.

We use $\mathcal {H}_\infty$, to denote the standard periodic Hilbert transform. Next, we introduce the periodic Hilbert transform on a strip.

#### Definition 3

(Periodic Hilbert transform on a strip) For $d<\infty$, we define the periodic Hilbert transform on $\mathbb {S}_d$, $\mathcal {H}_d:L^2_{2\pi }\rightarrow L^2_{2\pi }$ by its action on the orthonormal basis $\{\exp (inx)\}_{n\in \mathbb {Z}}$, as follows:

$$\begin{aligned} \mathcal {H}_d(\exp (inx))={\left\{ \begin{array}{ll}-i\coth (nd)\exp (inx)& \text {if }n\ne 0,\\ 0& \text {if }n= 0, \end{array}\right. } \end{aligned}$$

where $\coth$ denotes the hyperbolic cotangent.

Using the properties of the Hilbert transform and Lemma 1, we can reformulate (3) and (4) as

5 $$\begin{aligned} \frac{1}{2}(1+\Omega (y+y\mathcal {H}_dy_\alpha -\mathcal {H}_d(yy_\alpha )))^2=(B-Gy)((1+\mathcal {H}_dy_\alpha )^2+y_\alpha ^2)\qquad \text {on }\beta =0. \end{aligned}$$

We refer to [7] for a thorough derivation of (5).

A solution of (5) gives rise to a solution of the physical problem provided that

$$\begin{aligned} z(\alpha +i0)=\alpha +(i+\mathcal {H}_d)(y)(\alpha +i0) \end{aligned}$$

is injective and $z_\alpha (\alpha +i0)\ne 0$ for all $\alpha \in \mathbb R$. In the finite depth case, *H* is determined via the inversion of the map *z*, this *H* is unique up to a shift of *D* in the vertical direction.

We introduce the variable $\zeta =\exp (-i(\alpha +i\beta ))$ and the domains $\mathbb {D}:=\{\zeta \in \mathbb {C}:| \zeta |< 1\}$ and

$$\begin{aligned} \mathbb {A}_d:= {\left\{ \begin{array}{ll} \{\zeta \in \mathbb {D}:|\zeta |>\exp (-d)\}& \text {if }d\ne \infty ,\\ \mathbb {D}& \text {if } d=\infty \end{array}\right. } \end{aligned}$$

and define $w:\mathbb {A}_d\rightarrow \mathbb {C}$ to be such that

6 $$\begin{aligned} z(\zeta )=i\log \zeta + w(\zeta ). \end{aligned}$$

This decomposition transforms (5) into

$$\begin{aligned} \left( B-G\Im (w)\right) =\frac{1}{2}\frac{\left( 1+\Omega (\Im w+\mathcal {Q}^d(w)\right) ^2}{|1-i\zeta w_\zeta |^2}\qquad \text {for }|\zeta |=1, \end{aligned}$$

where $\mathcal {Q}^d$ consists of a commutator term, namely

$$\begin{aligned} \mathcal {Q}^d(w(\zeta )):= \Im (w)\mathcal {H}_d\left( \Im (-i\zeta w_\zeta )\right) -\mathcal {H}_d\left( \Im w\Im (-i\zeta w_\zeta )\right) \qquad \text {for }|\zeta |=1. \end{aligned}$$

### Operator Formulation

We define the Banach spaces

$$\begin{aligned} X&:= \{w\in C^{3+a}(\partial \mathbb {D},\mathbb {C}):w(\overline{\zeta })=w(\zeta )\},\\ Y&:= \{w\in C^{2+a}(\partial \mathbb {D},\mathbb {R}):w(\overline{\zeta })=w(\zeta )\}. \end{aligned}$$

For a given $w\in X$ and constant *d*, we can extend *w* to a function $\tilde{w}$ that is harmonic on $\mathbb {A}_d$ and satisfies the following conditions:

(i) $$\tilde{w}=w$$ on $$\partial \mathbb {D}$$;
(ii) If $d\ne \infty$, $\tilde{w}$ is constant on the inner boundary of $\mathbb {A}_d$. Moreover, the constant is chosen so that the mean over the inner circle of $\mathbb {A}_d$ is equal to the mean over the outer circle of $\mathbb {A}_d$.

We can then use $\tilde{w}$ and its harmonic conjugate to define a holomorphic function on $\mathbb {A}_d$. Next, we introduce the map $E:X\times (0,\infty ]\rightarrow Y$ which assigns to each pair (*w*, *d*) the function that is holomorphic on $\mathbb {A}_d$ and whose imaginary part satisfies conditions (i) and (ii) when $d\in (0,\infty )$; in the case $d=\infty$. $E(w,\infty )$ on *D* with its imaginary part satisfying condition (i). We then define the subset

$$\begin{aligned} U&:=\left\{ w\in X: 1-i\zeta \frac{\partial E(w,\infty )}{\partial \zeta }\ne 0 \right\} \end{aligned}$$

and the operator $\mathcal {G}:U\times \mathbb {R}\times (-\infty ,\tfrac{1}{3})\times \mathbb {R}\rightarrow Y$

7 $$\begin{aligned} \mathcal {G}(w,G,a,l)= &   \frac{1}{2}\frac{\left( 1+\Omega (a)\left( w+\mathcal {Q}^\frac{1}{l^2}\left( E(w,\frac{1}{l^2})\right) \right) \right) ^2}{|1-i\zeta \partial _\zeta E(w,\frac{1}{l^2})|^2}\nonumber \\  &   -\left( B(a)-G w\right) \qquad \text {for }|\zeta |=1, \end{aligned}$$

where

$$\begin{aligned} \mathcal {Q}^\frac{1}{l^2}= {\left\{ \begin{array}{ll} \mathcal {Q}^\frac{1}{l^2}& \text {if }l\ne 0,\\ \mathcal {Q}^\infty & \text {if }l= 0 \end{array}\right. } \end{aligned}$$

and

8 $$\begin{aligned} \Omega (a):= \frac{1-a}{1-3a},\qquad B(a):=\frac{1}{2}\left( \frac{1+a}{1-3a}\right) ^2. \end{aligned}$$

A similar operator was studied by Hur & Wheeler [1]. The difference between our operator and theirs is the additional parameter *l*.

## Overhanging Waves over a Finitely Deep Flow

We define

9 $$\begin{aligned} w(a)(\zeta ):=-\frac{4i \sqrt{a}\zeta }{1+\sqrt{a} \zeta }\qquad \text {for }|\zeta |=1, \end{aligned}$$

while noting this is the form Hur and Wheeler’s exact solutions [9] take in our notation. These solutions are overhanging for $a\in (a_{\text {crit}},a_{\text {max}})$. Here $a_{\text {crit}}=(\sqrt{2}-1)^2$ can be explicitly computed by solving $x_\alpha (\alpha ,0;a):=\Re (i\log \zeta +w(a)(\zeta )=0$ and $a_{\text {max}}\approx \sqrt{0.454670016452010}$ was given in [1].

It follows from [1] that $\mathcal {G}(w(a),0,a,0)=0$ for all $a\in (0,\frac{1}{4})$. The partial Fréchet derivative of $\mathcal {G}$ can be computed to be

$$\begin{aligned} \begin{aligned} D_w\mathcal {G}[w,G,a,0]v\!=&\!\frac{1\!+\!\Omega (a)(w\!+\!\mathcal {Q}(E(w,\infty ))}{|1\!-\!i\zeta \partial _\zeta E(w,\infty )|^2}\Omega (a)(v\!+\!\mathcal {Q}_w(E(w,\infty ))E(v,\infty )\\&-\frac{(1+\Omega (a)(w+\mathcal {Q}(E(w,\infty )))^2}{|1-i\zeta \partial _\zeta E(w,\infty )|^4}\\&\cdot \Im \left( \overline{(1-i\zeta \partial _\zeta E(w,\infty ))}\zeta \partial _\zeta E(v,\infty ) \right) +Gv. \end{aligned} \end{aligned}$$

This resembles the Fréchet derivative of the operator for the infinite depth case. See the discussion following in [1, Theorem 3]. In fact, we have that $D_w\mathcal {G}[\Im (w(a)),0,a,0]$ coincides with $D_w\mathcal {F}[w(a),0,a]$ written in terms of the imaginary part of *w*(*a*) restricted to the unit circle. Here $\mathcal {F}$ denotes the operator defined in [1, Section 4]. This implies that $D_w\mathcal {G}[\Im (w(a)),0,a,0]$ is an isomorphism. By the Implicit Function Theorem, [1, Theorem 3] can be extended in the following way.

### Theorem 4

For each $a_0\in (0,\tfrac{1}{4})$, there exists $\epsilon >0$ and a continuous operator

$$\begin{aligned} W:(-\epsilon ,\epsilon )\times (a_0-\epsilon ,a_0+\epsilon )\times (-\epsilon ,\epsilon )\rightarrow U \end{aligned}$$

such that $W(0,a,0)=\Im (w(a))$ and

$$\begin{aligned} \mathcal {G}(W(G,a,l);G,a,l)=0. \end{aligned}$$

Moreover, there exists a $\delta >0$ such that for all $(G,a,l)\in (-\epsilon ,\epsilon )\times (a_0-\epsilon ,a_0+\epsilon )\times (-\epsilon ,\epsilon )$, the following statements are equivalent.

(i) $$\mathcal {G}(w;G,a,l)=0$$ and $$||w-\Im (w(a))||_X<\delta $$;
(ii) $$w=W(G,a,l)$$.

By the same argument as in [1], Theorem 4 implies the following two results:

### Theorem 5

(Overhanging waves) For all $a\in (a_{\text {crit}},a_{\text {max}})$, there exists an $\epsilon >0$, such that for all $G\in (-\epsilon ,\epsilon )$ and all *H* sufficiently large, there exists a solution of (3) and (4b) with $\Omega =\Omega (a)$ and $B=B(a)$ whose profile is overhanging.

### Theorem 6

(Touching waves) There exists an $\epsilon >0$, such that for all $G\in (-\epsilon ,\epsilon )$ and all *H* sufficiently large, there exists a solution of (3) and (4b) whose profile intersects itself tangentially, enclosing a small bubble of air.

We note that these results were mentioned in Section 5 of [1].

## Continuous Curve of Solutions

This section aims to demonstrate the existence of a continuous curve of gravity waves connecting laminar flow and touching waves. We start by stating our results in the first subsection. Subsequently, in the second subsection, we establish a uniform version of Theorem 4.

### Statement of Results

We will prove a uniform version of Theorem 4 in the second subsection, but before that, let us state the theorem.

#### Theorem 7

(Main Theorem) For all $\gamma \in (0,\tfrac{1}{8})$, there exists $\epsilon >0$ and a continuous operator

$$\begin{aligned} W:S\rightarrow U, \end{aligned}$$

where

$$\begin{aligned} S:=\bigg \{(G,a,l)\in (-\epsilon ,\epsilon )\times (-\epsilon ,\tfrac{1}{4}-\gamma ]\times (-\epsilon ,\epsilon ): G\ge \tilde{G}(a,l)\bigg \}. \end{aligned}$$

Here, $\tilde{G}$ is a continuous surjective map between a two-dimensional and a one-dimensional neighborhood of the origin satisfying $\tilde{G}(0,0)=0$. The operator satisfies the following three conditions:

(i) $$W(0,a,l)=\Im (w(a))$$;
(ii) $W(\tilde{G}(a,l),a,l)$ is a constant function of $\zeta$;
(iii) $$\mathcal {G}(W(G,a,l);G,a,l)=0$$.

Moreover, there exists a $\delta >0$ such that for all $(G,a,l)\in S$, the following statements are equivalent:

(i) $$\mathcal {G}(w;G,a,l)=0$$ and $$||w-\Im (w(a))||_X<\delta $$;
(ii) $$w=W(G,a,l)$$.

The solutions from Theorem 7 give rise to solutions of () and (), allowing us to state the following results.

#### Theorem 8

There exists an $\epsilon >0$ such that for all $G\in (-\epsilon ,\epsilon )$ fixed, there exists a continuous curve of solutions of (3) and (4a) between a laminar flow and a touching wave.

#### Corollary 9

(Breaking Waves) For every sufficiently small $\epsilon >0$ and $G\in (-\epsilon ,\epsilon )$ fixed, there exists a solution of (3) and (4a) whose profile is vertical at a point but never overhanging.

#### Theorem 10

There exists an $\epsilon >0$ such that for all $G\in (-\epsilon ,\epsilon )$ fixed and all *H* sufficiently large, there exists a continuous curve of solutions of (3) and (4b) between a laminar flow and a touching wave.

#### Corollary 11

(Finite depth breaking waves) For every sufficiently small $\epsilon >0$, $G\in (-\epsilon ,\epsilon )$ and all *H* sufficiently large, there exists a solution of (3) and (4b) whose profile is vertical at a point but never overhanging.

### Proof of Main Theorem

We start using compactness to establish a version of Theorem 7 for compact subsets of $(0,\tfrac{1}{4})$. Then, we compute constant solutions of the operator problem. Subsequently, we find a curve of parameters such that the Fréchet Derivative of the operator at every point along the curve has the same kernel as at the point (0, 0, 0). Next, we use a local bifurcation result to construct solutions near the point (0, 0, 0). Finally, we connect this curve of solutions with the solutions obtained using compactness.

#### Lemma 12

For all $\lambda \in (0,\tfrac{1}{8})$, there exist $\epsilon >0$ and a real analytic operator

$$\begin{aligned} W:(-\epsilon ,\epsilon )\times [\lambda ,\tfrac{1}{4}-\lambda ]\times (-\epsilon ,\epsilon )\rightarrow U \end{aligned}$$

such that $W(0,a,0)=\Im (w(a))$ and

$$\begin{aligned} \mathcal {G}(W(G,a,l);G,a,l)=0. \end{aligned}$$

Moreover, there exists a $\delta >0$ such that for all $(G,a,l)\in (-\epsilon ,\epsilon )\times [\lambda ,\tfrac{1}{4}-\lambda ]\times (-\epsilon ,\epsilon )$ the following statements are equivalent.

(i) $$\mathcal {G}(w;G,a,l)=0$$ and $$||w-\Im (w(a))||_X<\delta $$;
(ii) $$w=W(G,a,l)$$.

#### Proof

We start by applying Theorem 4 to each $a\in [\lambda , \tfrac{1}{4}-\lambda ]$, yielding an $\epsilon _a$ and a $\delta _a$ satisfying the conditions in the theorem. Afterward, we cover $[\lambda ,\tfrac{1}{4}-\lambda ]$ with $\bigcup _{a\in [\lambda ,\tfrac{1}{4}-\lambda ]}\bigg (a-\epsilon _a, a+\epsilon _a\bigg )$. By the Heine–Borel Theorem, we can take a finite subcover, meaning

$$\begin{aligned} \big [\lambda , \tfrac{1}{4}-\lambda \big ]\subset \bigcup _{k=1}^n\bigg (a_k-\epsilon _{a_k}, a_k+\epsilon _{a_k}\bigg ). \end{aligned}$$

Afterward, we define $\delta :=\min _{k=1,..,n}\delta _{a_k}$. Let

10 $$\begin{aligned} W^k:(-\epsilon _{a_k},\epsilon _{a_k})\times (a_k-\epsilon _{a_k},a_k+\epsilon _{a_k})\times (-\epsilon _{a_k},\epsilon _{a_k})\rightarrow U \end{aligned}$$

be the operator obtained from applying Theorem 4 to $a_k$. This operator satisfies

$$\begin{aligned} ||W^k(G,a,l)-\Im (w(a))||_X<\delta _{a_k},\qquad \text {for }|a-a_k|<\epsilon _{a_k},\quad |G|<\epsilon _{a_k}\text { and } |l|<\epsilon _{a_k}. \end{aligned}$$

Since $W^k$ is continuous, we can choose $\tilde{\epsilon }_{a_k}$ such that

$$\begin{aligned} ||W^k(G,a,l)-\Im (w(a))||_X<\delta ,\qquad \text {for }|a-a_k|<\epsilon _{a_k},\quad |G|<\tilde{\epsilon }_{a_k}\text { and } |l|<\tilde{\epsilon }_{a_k}. \end{aligned}$$

Then, we define

$$\begin{aligned} \epsilon :=\min _{k=1,..,n}\tilde{\epsilon }_{a_k}. \end{aligned}$$

Next, we define the operator $W:(-\epsilon ,\epsilon )\times [\lambda ,\tfrac{1}{4}-\lambda ]\times (-\epsilon ,\epsilon )\rightarrow U$ by

11 $$\begin{aligned} W(G,a,l):= W^k(G,a,l) \qquad \text {if } |a-a_k|<\epsilon _{a_k}, k=1,...,n. \end{aligned}$$

Let *a* satisfy $|a-a_j|<\epsilon _{a_j}$ and $|a-a_k|<\epsilon _{a_k}$ for some *j* and *k*, *G* satisfy $|G|<\epsilon$ and *l* satisfy $|l|<\epsilon$. Then (*G*, *a*, *l*) is in the domain of both $W^j$ and $W^k$. Since

$$\begin{aligned} ||W^i(G,a,l)-\Im (w(a))||_X<\delta \qquad \text {for }i=j,k, \end{aligned}$$

it follows from the uniqueness of Theorem 4 that $W^j(G,a,l)=W^k(G,a,l)$, and therefore *W* is well defined.

We turn our attention now to the bifurcation point, starting by computing the partial Fréchet derivative at (*w*(0), 0, 0, 0),

12 $$\begin{aligned} D_w\mathcal {G}[\Im (w(0));0,0,0]v=\Im (E(v,\infty )-\zeta E(v,\infty )_\zeta )=-\Im \Big (\sum _{n=0}^\infty (n-1)v_n\zeta ^n\Big ), \end{aligned}$$

which is not invertible. Note that the symmetry assumption imposed on *X* implies the coefficients $v_n$ are purely imaginary. In fact, it follows from (12) that the kernel and the complement of the range of $D_w\mathcal {G}[w(0);0,0,0]$ are both one-dimensional. The kernel is spanned by $i\zeta$, and the complement of its range is spanned by $\Re (\zeta )$.

The next step is to look for constant solutions of $\mathcal {G}(w;G,a,l)=0$, which correspond to laminar flow solutions of (3).

#### Lemma 13

There exists a real analytic operator $d:\mathbb {R}\times (-\infty ,1/3)\rightarrow \{iR:R\in \mathbb {R}\}$ such that

$$\begin{aligned} \mathcal {G}(d(G,a);G,a,l)=0\qquad \qquad \text {and}\qquad \qquad d(0,0)=0 \end{aligned}$$

for $(G,a)\in \mathbb {R}\times (-\infty ,1/3)$ and $l\in \mathbb {R}$. Moreover, there exist open neighborhoods $V\subset \mathbb {R}^2$, $T\subset \mathbb {R}$ containing zero and a smooth map $\tilde{G}:V\rightarrow T$, such that

$$\begin{aligned} \ker D_x\mathcal {G}\Big [d(\tilde{G}(a,l),a); \tilde{G}(a,l), a,l\Big ]=\langle i\zeta \rangle _X \end{aligned}$$

for $(a,l)\in V$, $\tilde{G}(0,0)=0$ and for every fixed *l*, the map $\tilde{G}_l(a):= \tilde{G}(a,l)$ is invertible.

#### Proof

Let *c* be a fixed constant. A computation reveals

$$\begin{aligned} \mathcal {G}(ic;G,a,l)=\frac{1}{2}(1+\Omega (a)c)^2+Gc-B(a). \end{aligned}$$

Thus, $\mathcal {G}(ic;G,a,l)=0$ if and only if

13 $$\begin{aligned} c=-\frac{\Omega (a)+G}{\Omega (a)^2}\pm \frac{1}{\Omega (a)^2}\sqrt{G^2+2\Omega (a)G+2 B(a)\Omega (a)^2}. \end{aligned}$$

Evaluating this expression at $a=G=0$, we find that when the sign before the square root is positive, $c=0$. We define the operator $d:\mathbb {R}\times (-\infty ,1/3)\rightarrow \{iR:R\in \mathbb {R}\}$ by

14 $$\begin{aligned} d(G,a)=i\bigg (-\frac{\Omega (a)+G}{\Omega (a)^2}+\frac{1}{\Omega (a)^2}\sqrt{G^2+2\Omega (a)G+2 B(a)\Omega (a)^2}\bigg ). \end{aligned}$$

We turn our focus to the second part of the lemma and compute the Fréchet derivative of (7) at (*d*(*G*, *a*); *G*, *a*, *l*),

15 $$\begin{aligned} \begin{aligned} D_w\mathcal {G}[d(G,a); G, a,l]v=&\,\Im \bigg (\Big ((1+\Omega (a)d(G,a))\Omega (a)+G\Big )E(v,\frac{1}{l^2}) \\  &-(1+\Omega (a)d(G,a))^2\zeta \partial _\zeta E(v,\frac{1}{l^2})\bigg ). \end{aligned} \end{aligned}$$

By plugging in $v=i\zeta ^k$ in (15) and equating it to zero, we obtain

$$\begin{aligned} \big ((1 + \Omega (a) \Im (d(G,a)))\Omega (a) + G\big )E(v, \frac{1}{l^2})-k(1 + \Omega (a) \Im (d(G,a)))^2 E(v,\frac{1}{l^2})=0. \end{aligned}$$

For sufficiently small *a*, the left-hand side of () is a strictly monotonous function of *k*. This implies that $i\zeta$ spans the kernel of $D_w\mathcal {G}[d(G,a); G, a,l]$ if and only if

16 $$\begin{aligned} \big ((1 + \Omega (a) \Im (d(G,a)))\Omega (a) + G\big )E(v,\frac{1}{l^2})-(1 + \Omega (a) \Im (d(G,a)))^2 E(v,\frac{1}{l^2})=0. \end{aligned}$$

We note that in a neighborhood of (0, 0, 0), (14) can be described in the following way,

17 $$\begin{aligned} \Im (d(G,a))=4a(1+\mathcal {O}(a+G)). \end{aligned}$$

Therefore, we can compute the partial derivative with respect to *G* of the left-hand side of (16) evaluated at $(G,a,l)=(0,0,0)$ to be equal to 1. An application of the Implicit Function Theorem yields $\tilde{G}$ as described in the lemma. Fixing *l* and applying the Implicit Function Theorem with respect to *a* will yield the invertibility claim.

#### Lemma 14

There exist $\epsilon >0$ and a smooth operator $v:\tilde{S}\rightarrow U$, where

$$\begin{aligned} \tilde{S}:= \left\{ (G,a,l)\in \mathbb {R}\times (-\epsilon ,\epsilon )\times (-\epsilon ,\epsilon ):\tilde{G}(a,l)\le G< \tilde{G}(a+\epsilon ,l+\epsilon )\right\} \end{aligned}$$

such that *v*(*G*, *a*, *l*) satisfies the equation

$$\begin{aligned} \mathcal {G}\left( v(G,a,l)+d(G,a);G,a,l\right) =0 \end{aligned}$$

for $(G,a,l)\in \tilde{S}$. Moreover, *v*(*G*, *a*, *l*) is of the form

18 $$\begin{aligned} v(G,a,l)=C_{G,l}(a-\tilde{G}_l^{-1}(G))^\frac{1}{2}\Re \zeta +\mathcal {O}((a-\tilde{G}_l^{-1}(G))+l), \end{aligned}$$

where $C_{G,l}$ is a positive constant that depends continuously on *G* and *l*. Furthermore, there exists a $\delta >0$ such that $v(G,a,l)+d(G,a)$ is the only non-trivial solution of $\mathcal {G}\big (v,G,a,l\big )=0$, satisfying $||v||_X<\delta$.

#### Proof

We start by defining the auxiliary operator $\mathcal {K}:U\times \mathbb {R}\times (-\infty ,1/3)\times \mathbb {R}\rightarrow Y$ by

19 $$\begin{aligned} \mathcal {K}(v;G,a,l)=\mathcal {G}( d(G,a)+v;G,a,l). \end{aligned}$$

It follows that $\mathcal {K}(0;G,a,l)=0$, for all $(G,a,l)\in \mathbb {R}\times (-\infty ,\tfrac{1}{3})\times \mathbb {R}$. Moreover,

$$\begin{aligned}  &   \ker D_v\mathcal {K}\left( 0;\tilde{G}(a_{0},l_0),a_0,l_0)\right) =\langle i\zeta \rangle _X\quad \text {and}\\  &   \quad {{\,\textrm{ran}\,}}D_v\mathcal {K}\left( 0;\tilde{G}(a_{0},l_0),a_0,l_0\right) =Y\ominus \langle \Re (\zeta )\rangle _Y \end{aligned}$$

for all $(a_0,l_0)\in V$. Next, we compute the mixed derivative

20 $$\begin{aligned} D^2_{v a}\mathcal {K}\left( 0;\tilde{G}(a_{0},0),a_0,0\right) (i\zeta )=\Big (-2+\mathcal {O}(a_0)\Big )\Re \zeta . \end{aligned}$$

Varying *l* in a neighborhood of 0 will only create smooth dependence on *l* (see (A.2) and the discussion following it in [17]), the same is true for the other partial derivatives below, so without loss of generality, we consider $l=0$. The expression (20) needs to be nonzero in order to satisfy the transversality condition. To achieve this, we redefine *V* if necessary. Then, we can apply Corollary 17 to obtain real analytic operators

21 $$\begin{aligned} \tilde{v}:(-\tilde{\epsilon },\tilde{\epsilon })^3\rightarrow U\qquad \text {and}\qquad \tilde{a}:(-\tilde{\epsilon },\tilde{\epsilon })^3\rightarrow \mathbb {R}, \end{aligned}$$

such that

$$\begin{aligned} \mathcal {K}\Big (\tilde{v}(s,G,l),G,\tilde{a}(s,G,l),l\Big )=0, \text {for all }|s|,|G|,|l|<\tilde{\epsilon }, \end{aligned}$$

$\tilde{v}(s,G,l)=si\zeta +\mathcal {O}(s^2+sG+sl)$. We can then use the bifurcation formulas from Sect. I.6 of [18] to compute the derivatives of $\tilde{a}(s,G,l)$ with respect to *s*. We proceed with computing the second-order Fréchet derivative at $(0,\tilde{G}(a_0,0),a_0,0)$,

22 $$\begin{aligned}&D^2_{vv}\mathcal {K}(0,\tilde{G}(a_0,0),a_0,0)(u)^2= \Omega (a_0)\mathcal {Q}_{ww}(d(\tilde{G}(a_0,0),a))E(u,0)^2\nonumber \\&\quad -4\Omega (a_0)\Im (E(u,0))\Im (\zeta E(u,0)_\zeta )+4\Big (\Im (\zeta E(u,0)_\zeta )\Big )^2\\&\quad -\Im (i|E(u,0)_\zeta |^2),\nonumber \end{aligned}$$

where we use $\mathcal {Q}_{w}(d(G,a))(u)=0$. Evaluating (22) at $u=i\zeta$ reveals

23 $$\begin{aligned} D^2_{vv}\mathcal {K}(0,\tilde{G}(a_0),a_0,0)(i\zeta )^2=\Omega (a_0)-4\Omega (a_0)\Re (\zeta )^2+4\Re (\zeta )^2-1, \end{aligned}$$

where we used $\mathcal {Q}_{w}(d(G,a))(v)=0$ and $\mathcal {Q}_{ww}(d(G,a))(i\zeta )^2=1$. Note that (23) has zero projection on $\langle \Re (\zeta )\rangle _Y$ and therefore, $\tfrac{\partial }{\partial s}\tilde{a}(0,\tilde{G}(a_0))=0$. Subsequently, we compute the third-order linearization in *v* at $(0,\tilde{G}(a_0),a_0)$,

24 $$\begin{aligned} D^3_{vvv}\mathcal {K}(0,\tilde{G}(a_0),a_0,0)(u)^3&=3\Omega (a_0)^2(\Im u)\mathcal {Q}_{ww}(u)^2\nonumber \\&-6\Omega (a_0)\mathcal {Q}_{ww}(u)^2\Im (\zeta u_\zeta )-6\Omega (a_0)^2(\Im u)^2\Im (\zeta u_\zeta )\end{aligned}$$

25 $$\begin{aligned}&+3\Omega (a_0)\Im (u)\bigg (8\Big (\Im (\zeta u_\zeta )\Big )^2-2\Im (i|u_\zeta |^2)\bigg )\\&-24\Big (\Im (\zeta u_\zeta )\Big )^3+12\Im (\zeta u_\zeta )\Im (i|u_\zeta |^2).\nonumber \end{aligned}$$

Evaluating (24) at $u=i\zeta$ reveals

$$\begin{aligned} D^3_{vvv}\mathcal {K}(0,\tilde{G}(a_0),a_0,0)(i\zeta )^3=3\big (\Omega (a_0)-2\big )^2\Re \zeta -6(\Omega (a_0)-2)^2(\Re \zeta )^3, \end{aligned}$$

which has nonzero projection on $\langle \Re (\zeta )\rangle _Y$. It follows that $\tfrac{\partial ^2}{\partial s^2}\tilde{a}(0,\tilde{G}(a_0),0)=\tfrac{(\Omega (a_0)-2)^2}{2+\mathcal {O}(a_0)}$. We, again, possibly, redefine *V* to ensure $\tfrac{\partial ^2}{\partial s^2}\tilde{a}(0,\tilde{G}(a_0),0)>0$, for all $a_0\in V$. Since $\tfrac{\partial ^2}{\partial s^2}\tilde{a}(0,\tilde{G}(a_0),0)>0$, it follows that we can choose an $\epsilon$ such that the real analytic operator $\tilde{a}(\cdot ,\tilde{G}(a_0),0)$ is injective on the set $[0,\epsilon )$. We define $v:(-\epsilon ,\epsilon )\times [a_0,a_0+\epsilon )\times (-\epsilon ,\epsilon )\rightarrow U$ by

$$\begin{aligned} v(G,a,l):= \tilde{v}\left( \tilde{a}^{-1}(s,G,l)\right) . \end{aligned}$$

The formula in (18) follows directly from the definition above.

#### Proof of Theorem 7

Let $\gamma >0$ be given. Let $v,\epsilon _1,\delta _1$ and $\tilde{S}$ be as described in Lemma 14. Next, we note that for small and positive *a*, (9) can be expressed as

26 $$\begin{aligned} w(a)=-\frac{4i\sqrt{a}\zeta }{1+\sqrt{a}\zeta }=-4i\sqrt{a}\zeta +\mathcal {O}(a). \end{aligned}$$

Therefore, we can choose a positive and small enough $\lambda$ such that $||w(\lambda )||_X<\tfrac{\delta _1}{2}$, $||d(0,\lambda )||_X<\tfrac{\delta _1}{2}$, $\lambda <\epsilon _1$ and $\lambda <\gamma$. We then apply Lemma 12 for such a $\lambda$, which yields $\epsilon _2,\delta _2$ and $W^{\lambda }$ as described in the lemma. We then define

$$\begin{aligned} \delta :=\min (\delta _1,\delta _2). \end{aligned}$$

We choose $\tilde{\epsilon }_1$ and $\tilde{\epsilon }_2$ to be small enough such that

$$\begin{aligned} ||v(G,a,l)-d(G,a)||_X<\delta \qquad \text { for }|G|,|l|<\tilde{\epsilon }_1 \end{aligned}$$

and

$$\begin{aligned} ||W^\lambda (G,a)-w(a)||_X<\delta \qquad \text { for }|G|,|l|<\tilde{\epsilon }_2. \end{aligned}$$

We then define

$$\begin{aligned} \epsilon := \min (\tilde{\epsilon }_1,\tilde{\epsilon }_2) \end{aligned}$$

and

$$\begin{aligned} S:=\bigg \{(G,a)\in (-\epsilon ,\epsilon )\times (-\infty ,\tfrac{1}{\sqrt{2}}-\gamma ]:a\ge \tilde{G}^{-1}(G)\bigg \}. \end{aligned}$$

Let $W:S\rightarrow U$ be defined by

27 $$\begin{aligned} W(G,a):= {\left\{ \begin{array}{ll} W^\lambda (G,a) &  \text {if } a>\lambda \\ v(G,a)+d(G,a) &  \text {if }(G,a)\in \tilde{S}\cap S. \end{array}\right. } \end{aligned}$$

Next, we show (27) is well-defined. Let $(G,a)\in \tilde{S}$ and $a>\lambda$, then

$$\begin{aligned} \mathcal {K}\Big (W_\lambda (G,a)-d(G,a),G,a\Big )=0 \end{aligned}$$

and

$$\begin{aligned} ||W_\lambda (G,a)-d(G,a)||_X<\frac{\delta (\mu )}{2}+\frac{\delta (\mu )}{2}\le \delta (\mu ). \end{aligned}$$

By Lemma 14, the solutions coincide, meaning (27) is well-defined.

## Critical Layers

Critical layers are defined as the set of points where the horizontal velocity,

28 $$\begin{aligned} \psi _y=\frac{\psi _\alpha y_\alpha +\psi _\beta x_\alpha }{|z_\alpha |^2} \end{aligned}$$

vanishes. Our next result characterizes the local behavior of a critical layer when it intersects at a point on the surface where the tangent is vertical but which is not a stagnation point. Notably, the results hold for a general water wave and not just the ones we constructed.

### Proposition 15

Let *z* denote the conformal map to the half plane of a solution to (3) with $\tau =0$ and assume the same conditions as in Proposition 2. Additionally, assume that $\alpha _{\text {crit}}$ is a point in the $(\alpha ,\beta )$ plane at which the wave profile is vertical.

(i) If the function $x(\alpha +i0)$ does not have a local extremum at $\alpha _{\text {crit}}$ and $G>0$, then no critical layers touch the free surface at $\alpha _{\text {crit}}$. However, their analytic extension from outside the fluid domain do touch $\alpha _{\text {crit}}$;
(ii) If the function $x(\alpha +i0)$ does not have a local extremum at $\alpha _{\text {crit}}$ and $G<0$, there exist critical layers from the water touching the point $\alpha _{\text {crit}}$. Moreover, their analytic extension from outside the fluid domain do not touch $\alpha _{\text {crit}}$;
(iii) If the function $x(\alpha +i0)$ has a local extremum at $\alpha _{\text {crit}}$ and $G\ne 0$, there exist critical layers from the water touching the point $\alpha _{\text {crit}}$. Moreover, their analytic extension from outside the fluid domain also touch $\alpha _{\text {crit}}$.

### Proof

Without loss of generality, assume the wave is traveling in the positive *x* direction. Let $\alpha _{\text {crit}}$ denote the point at which the wave is vertical. It follows that

$$\begin{aligned} x_\alpha (\alpha _{\text {crit}},0)=0 \end{aligned}$$

and the smallest positive integer *k*, such that $\frac{\partial ^k}{\partial \alpha ^k}x(\alpha _{\text {crit}},0)$ does not vanish, must be even if the wave is a breaking wave and odd if the wave overhangs at the point. Moreover

29 $$\begin{aligned} \frac{\partial ^k}{\partial \alpha ^k}x(\alpha _{\text {crit}},0) >0 \end{aligned}$$

if *k* is odd. It follows from (28) that

30 $$\begin{aligned} \psi _y=0\iff \psi _\alpha y_\alpha +\psi _\beta x_\alpha =0. \end{aligned}$$

Let *U* be the open set from Proposition 2 where $\psi$ and *z* have a real analytic extension and define $F:U\rightarrow \mathbb {R}$ by

31 $$\begin{aligned} F(\alpha ,\beta )=\psi _\alpha (\alpha ,\beta ) y_\alpha (\alpha ,\beta )+\psi _\beta (\alpha ,\beta )x_\alpha (\alpha ,\beta ). \end{aligned}$$

(30) implies $(\alpha ,\beta )$ belongs to the critical layer if and only if $F(\alpha ,\beta )=0$. Since $F(\alpha _\text {crit},0)=0$, we want to use Taylor expansions of (31) near the point $(\alpha _\text {crit},0)$ to figure out the local behaviour of the critical layers.

Notice that $\psi$ is constant on $\{\beta =0\}$, and therefore

32 $$\begin{aligned} \frac{\partial ^j}{\partial \alpha ^j}\psi (\alpha _\text {crit},0)=0,\text { for all }j\in \mathbb {Z}^+. \end{aligned}$$

Next, we rewrite (3c) as

33 $$\begin{aligned} \psi _\beta =\pm \sqrt{2(x_\alpha ^2+y_\alpha ^2)(B-G y)}\qquad \text {on }\{\beta =0\} \end{aligned}$$

for later use. Given all the $\alpha$ derivatives of $\psi$ vanish at the critical point, exploring alternative branches of (33) results in the partial derivatives of *F* at the critical point differing only in sign. Consequently, we can consider the positive branch of Eq. (33) without any loss of generality.

We proceed to compute the partial derivatives of *F* at the point $(\alpha _\text {crit},0)$. Starting by

$$\begin{aligned} \begin{aligned} \frac{\partial ^j}{\partial \alpha ^j}F(\alpha ,\beta )=\sum _{l=0}^j\left( {\begin{array}{c}j\\ l\end{array}}\right) \left( \frac{\partial ^l\psi _\alpha }{\partial \alpha ^l}\frac{\partial ^{j-l}y_\alpha }{\partial \alpha ^{j-l}}+\frac{\partial ^l\psi _\beta }{\partial \alpha ^l}\frac{\partial ^{j-l}x_\alpha }{\partial \alpha ^{j-l}}\right) ,\text { for all }j\in \mathbb {Z}^+. \end{aligned} \end{aligned}$$

Using (32) and $\frac{\partial ^j}{\partial \alpha ^j}x=0$ for $j<k$ to evaluate the expression above at $(\alpha _{\text {crit}},0)$ yields

34 $$\begin{aligned} \frac{\partial ^j}{\partial \alpha ^j}F(\alpha _{\text {crit}},0)={\left\{ \begin{array}{ll}0,& \text {if }j<k-1, \\ \psi _\beta \frac{\partial ^kx}{\partial \alpha ^k},& \text {if }j=k-1. \end{array}\right. } \end{aligned}$$

Next, we compute the derivatives with respect to $\beta$

35 $$\begin{aligned} \begin{aligned} \frac{\partial ^j}{\partial \beta ^j}F(\alpha ,\beta )&=\sum _{l=0}^j\left( {\begin{array}{c}j\\ l\end{array}}\right) \left( \frac{\partial ^l\psi _\alpha }{\partial \beta ^l}\frac{\partial ^{j-l}y_\alpha }{\partial \beta ^{j-l}}+\frac{\partial ^l\psi _\beta }{\partial \beta ^l}\frac{\partial ^{j-l}x_\alpha }{\partial \beta ^{j-l}}\right) .\\ \end{aligned} \end{aligned}$$

Plugging in $(\alpha _{\text {crit}},0)$ for $j=1$ above yields

36 $$\begin{aligned} \begin{aligned} \frac{\partial }{\partial \beta }F(\alpha _\text {crit},0)&=\psi _{\alpha \beta }y_\alpha -\psi _\beta y_{\alpha \alpha }\\&=\left( \sqrt{2(B-G y)}y_{\alpha \alpha }-\frac{G y_\alpha ^2 }{\sqrt{2(B-G y)}}\right) y_\alpha -\sqrt{2(B-G y)}y_\alpha y_{\alpha \alpha }\\  &=-\frac{G y_\alpha ^3 }{\sqrt{2(B-G y)}}. \end{aligned} \end{aligned}$$

We utilized Eq. (33) above to calculate the derivatives of $\psi _\beta$. Additionally, we assume $\sqrt{y_\alpha ^2}=y_\alpha$ because symmetry implies the existence of a critical point where $y_\alpha$ is positive and another where it is negative. The Taylor expansion of *F* at the point $(\alpha _\text {crit},0)$ is then

37 $$\begin{aligned} \begin{aligned}&\left( -\frac{G y_\alpha ^3 }{\sqrt{2(B-G y)}}\right) \Bigg |_{\alpha =\alpha _{\text {crit}}}\beta +\left( \sqrt{2(B-G y)} y_\alpha \frac{\partial ^kx}{\partial \alpha ^k}\right) \Bigg |_{\alpha =\alpha _{\text {crit}}}(\alpha -\alpha _{\text {crit}})^{k-1}\\=&\mathcal {O}\Big (|\alpha -\alpha _{\text {crit}}|^k+|\beta |^2+|(\alpha -\alpha _{\text {crit}})\beta |\Big ). \end{aligned} \end{aligned}$$

When *k* is odd, (37) has the form

38 $$\begin{aligned} -C_1 G \beta +C_2(\alpha -\alpha _{\text {crit}})^{k-1}=\mathcal {O}\Big (|\alpha -\alpha _{\text {crit}}|^k+|\beta |^2+|(\alpha -\alpha _{\text {crit}})\beta |\Big ), \end{aligned}$$

where $C_1$ and $C_2$ denote positive constants that depend on *G*. It follows from applying the Implicit Function Theorem to (38) that solutions of $F(\alpha ,\beta )=0$ in a neighborhood of $(\alpha _\text {crit},0)$ have the form

39 $$\begin{aligned} \beta =\frac{C_2}{C_1 G} (\alpha -\alpha _\text {crit})^{k-1} + \mathcal {O}( |\alpha -\alpha _\text {crit}|^{k}). \end{aligned}$$

Since the exponent $k-1$ is even, statements (*i*) and (*ii*) follow. When *k* is even, (37) has the form

40 $$\begin{aligned} C_3 G \beta +C_4(\alpha -\alpha _{\text {crit}})^{k-1}=\mathcal {O}\Big (|\alpha -\alpha _{\text {crit}}|^k+|\beta |^2+|(\alpha -\alpha _{\text {crit}})\beta |\Big ), \end{aligned}$$

where $C_3$ and $C_4$ denote constants that depend on *G*. It follows from applying the Implicit Function Theorem to (40) that solutions of $F(\alpha ,\beta )=0$ in a neighborhood of $(\alpha _\text {crit},0)$ have the form

41 $$\begin{aligned} \beta =\frac{C_4}{C_3 G} (\alpha -\alpha _\text {crit})^{k-1} + \mathcal {O}( |\alpha -\alpha _\text {crit}|^{k}). \end{aligned}$$

Since the exponent $k-1$ is odd, statement (*iii*) follows:

## Data Availability

No datasets were generated or analysed during the current study.

## Appendix A Local Bifurcation Theory

In this appendix, we provide the local bifurcation theory used throughout the paper. The proposition below is a modified version of the Crandall–Rabinowitz Theorem [19, Theorem 8.3.1] with two additional parameters. The proof is very similar to that in the reference.

### Proposition 16

Let *X* and *Y* be Banach spaces, $\mu _0,\lambda _0,\nu _0\in \mathbb {R}$ and $F:X\times \mathbb {R}\times \mathbb {R}\times \mathbb {R}\rightarrow Y$ an analytic operator satisfying the following three properties:

(i) $$F(0,\mu ,\lambda ,\nu )=0$$, for all $$\mu ,\lambda ,\nu \in \mathbb {R}$$;
(ii) $D_x F[0, \mu _0,\lambda _0,\nu _0]$ is a Fredholm operator of index zero with a one-dimensional kernel spanned by $x_0\in X$;
(iii) $D_{x \mu }F[0,\mu _0,\lambda _0,\nu _0](x_0)\not \in {{\,\textrm{ran}\,}}(D_x F[0, \mu _0,\lambda _0,\nu _0])$ (transversality condition).

Then there exist $\epsilon >0$ and a curve $(\chi (s,\lambda ,\nu ),\mu (s,\lambda ,\nu ),\lambda ,\nu )$, where

$$\begin{aligned} \chi (s,\lambda ,\nu )&=s x_0+\mathcal {O}\left( s^2+s(\lambda -\lambda _0)+s(\nu -\nu _0)\right) \\\mu (s,\lambda ,\nu )&=\mu _0 +\mathcal {O}\left( s+(\lambda -\lambda _0)+(\nu -\nu _0)\right) ,\end{aligned}$$

such that $F(\chi (s,\lambda ,\nu );\mu (s,\lambda ,\nu ),\lambda ,\nu )=0$ for $s,(\lambda -\lambda _0),(\nu -\nu _0)\in (-\epsilon ,\epsilon )$. In addition, there exists a neighborhood of $(0,\mu _0,\lambda _0,\nu _0)$ where these are the only non-trivial solutions.

### Corollary 17

Assuming the same conditions as stated in Proposition 16, and further, that there exists a continuous curve of parameters $S:=\{(\mu (t),\lambda (t),\nu (t)):t\in (-\delta ,\delta )\}$, satisfying the following four properties:

(i) $$(\mu (0),\lambda (0),\nu (0))=(\mu _0,\lambda _0,\nu _0)$$;
(ii) For all $t\in (-\delta ,\delta )$, $D_x F[0, \mu (t),\lambda (t),\nu (t)]$ is a Fredholm operator of index zero with one-dimensional kernel spanned by $x_0\in X$;
(iii) For all $t\in (-\delta ,\delta )$, $D_{x \mu }F[0,\mu (t),\lambda (t),\nu (t)](x_0)\not \in {{\,\textrm{ran}\,}}(D_x F[0, \mu _0,\lambda _0,\nu _0])$ (transversality condition)
(iv) The mapping $t\mapsto D_{x \mu }F[0,\mu (t),\lambda (t),\nu (t)](x_0)$ is continuous.

Then, there exists $\epsilon >0$ and a curve $(\chi (s,\lambda ,\nu ),\mu (s,\lambda ,\nu ),\lambda ,\nu )$, where

$$\begin{aligned} \chi (s,\lambda ,\nu )&=s x_0+\mathcal {O}\left( s^2+s(\lambda -\lambda _0)+s(\nu -\nu _0)\right) ,\\\mu (s,\lambda ,\nu )&=\mu _0 +\mathcal {O}\left( s+(\lambda -\lambda _0)+(\nu -\nu _0)\right) \end{aligned}$$

such that $F(\chi (s,\lambda ,\nu );\mu (s,\lambda ,\nu ),\lambda ,\nu )=0$ for $s,(\lambda -\lambda _0),(\nu -\nu _0)\in (-\epsilon ,\epsilon )$.

Moreover, for every fixed $(\lambda (t),\nu (t))\in (\lambda _0-\epsilon ,\lambda _0+\epsilon )\times (\nu _0-\epsilon ,\nu _0+\epsilon )$, the curve

$$\begin{aligned} (\chi (s,\lambda (t),\nu (t)),\mu (s,\lambda (t),\nu (t))\end{aligned}$$

is the same as the curve obtained by applying the Crandall–Rabinowitz Theorem at the point

$$\begin{aligned} (0,\mu (t),\lambda (t),\nu (t)).\end{aligned}$$

In addition, there exists a neighborhood of $(0,\mu _0,\lambda _0,\nu _0)$ where these are the only non-trivial solutions.

The statement above follows from applying the Proposition 16 at every point along the parameter curve while keeping $\lambda$ fixed, and then shrinking the neighborhoods so that the uniqueness from Proposition 16 implies that the new solutions are the same as those obtained from Proposition 16.
